# Brain Functional Connectivity Is Different during Voluntary Concentric and Eccentric Muscle Contraction

**DOI:** 10.3389/fphys.2016.00521

**Published:** 2016-11-15

**Authors:** Wan X. Yao, Zhiguo Jiang, Jinqi Li, Changhao Jiang, Crystal G. Franlin, Jack L. Lancaster, Yufei Huang, Guang H. Yue

**Affiliations:** ^1^Department of Kinesiology, Health, and Nutrition, University of Texas at San AntonioSan Antonio, TX, USA; ^2^Human Performance and Engineering Research, Kessler FoundationWest Orange, NJ, USA; ^3^Research Imaging Center, University of Texas Health Science Center at San AntonioSan Antonio, TX, USA; ^4^Beijing Key Lab of Physical Fitness Evaluation and Tech Analysis, Capital University of Physical Education and SportsBeijing, China

**Keywords:** brain functional connectivity, concentric contraction, eccentric contraction, fMRI

## Abstract

Previous studies report greater activation in the cortical motor network in controlling eccentric contraction (EC) than concentric contraction (CC) of human skeletal muscles despite lower activation level of the muscle associated with EC. It is unknown, however, whether the strength of functional coupling between the primary motor cortex (M1) and other involved areas in the brain differs as voluntary movements are controlled by a network of regions in the primary, secondary and association cortices. Examining fMRI-based functional connectivity (FC) offers an opportunity to measure strength of such coupling. To address the question, we examined functional MRI (fMRI) data acquired during EC and CC (20 contractions each with similar movement distance and speed) of the right first dorsal interosseous (FDI) muscle in 11 young (20–32 years) and healthy individuals and estimated FC between the M1 and a number of cortical regions in the motor control network. The major findings from the mechanical and fMRI-based FC analysis were that (1) no significant differences were seen in movement distance, speed and stability between the EC and CC; (2) significantly stronger mean FC was found for CC than EC. Our finding provides novel insights for a better understanding of the control mechanisms underlying voluntary movements produced by EC and CC. The finding is potentially helpful for guiding the development of targeted sport training and/or therapeutic programs for performance enhancement and injury prevention.

## Introduction

Our daily movements are largely produced by shortening (concentric) and lengthening (eccentric) muscle contractions. In the past, there has been considerable interest in identifying differential control mechanisms underlying the lengthening and shortening contractions due to observations of different neuromuscular and biomechanical outcomes as the consequence of the two types of contractions. For example, following a given level of muscle activation, the force generated by an eccentric contraction (EC) is greater than that by a concentric contraction (CC). A fundamental question raised is if a CC requires a different neural control strategy from the one used by an EC of the same muscle. There has been indirect evidence suggesting unique control strategies for CC and EC by past studies. For example, the studies by Howell et al. (1995) and Nardone et al. (1988) suggest that EC and CC follow different motor-unit recruitment orders during non-fatigue muscle contractions. However, contradictory findings showing that EC and CC follow the same motor-unit recruitment orders have also been reported (Bawa and Jones, 1999; Stotz and Bawa, 2001) for the contradictory findings which show that EC and CC follow the same motor-unit recruitment orders. In addition, Kossev and Christova (1998) found that in contrast to the concentric movements, the eccentric movement is associated with a significant amount of rate coding, which also indicates differences in the muscle force control between CC and EC. Furthermore, compared to CC, EC had a smaller magnitude of electromyographic (EMG) signal against a given resistance, and depressed corticospinal neuron (Abbruzzese et al., 1994; Sekiguchi et al., 2001) and monosynaptic reflex (Moritani et al., 1988; Abbruzzese et al., 1994) excitability. Studies by Duclay and colleagues postulate that the differences in EMG activities between the two types of contractions are a result of differential modulations of motoneuron excitability at supraspinal and/or spinal levels (Duclay and Martin, 2005; Duclay et al., 2011), and the modulation of the spinal motoneuron excitability by the supraspinal centers can be contraction-type specific (Duclay et al., 2009). In an attempt to delineate potential differential control mechanisms at the cortical level between voluntary EC and CC, Yao et al. (2014) examined functional MRI (fMRI) data acquired during EC and CC of the first dorsal interosseous (FDI) muscle in both young and old healthy individuals. They found that the EC resulted in significantly stronger activation in the motor control network consisting of primary, secondary and association motor cortices than CC in the young and old groups; and that in the primary motor and sensory cortices, the biased activation toward EC was significantly greater in the young than the old groups.

It should be noted that although Yao et al. (2014) provided more detailed evidence showing brain regions involved in controlling the CC and EC activities and differences in cortical involvements when controlling the CC and EC, it is unknown if the strength of functional coupling between the primary motor cortex and other areas in the motor control network differs. Addressing this question is important for a more comprehensive understanding central nervous system strategies for controlling human voluntary movements that are consisted of CC and EC. Examining fMRI-based functional connectivity (FC) offers an opportunity to measure strength of such coupling. Thus, the purpose of this study was to examine FC within cortical motor control network based on fMRI data collected during CC and EC contractions. It was hypothesized that both contraction types would evoke brain activation in a similar network (with varied amplitude; Yao et al., 2014) but the strength (intensity) of FC between the left primary motor cortex and other activated regions would be different.

## Methods

### Subjects

Eleven subjects (4 males and ranging from 20 to 32 years) participated in the study. All subjects were right-handed determined by the Edinburgh inventory (Oldfield, 1971) without any neurological, neuromuscular and musculoskeletal impairments. The study was approved by the Institutional Review Boards of the University of Texas at San Antonio and the University of Texas Health Science Center at San Antonio. Written informed consents were obtained from all the subjects prior to their participation.

### Tasks and experimental setup

**Figure 1:**
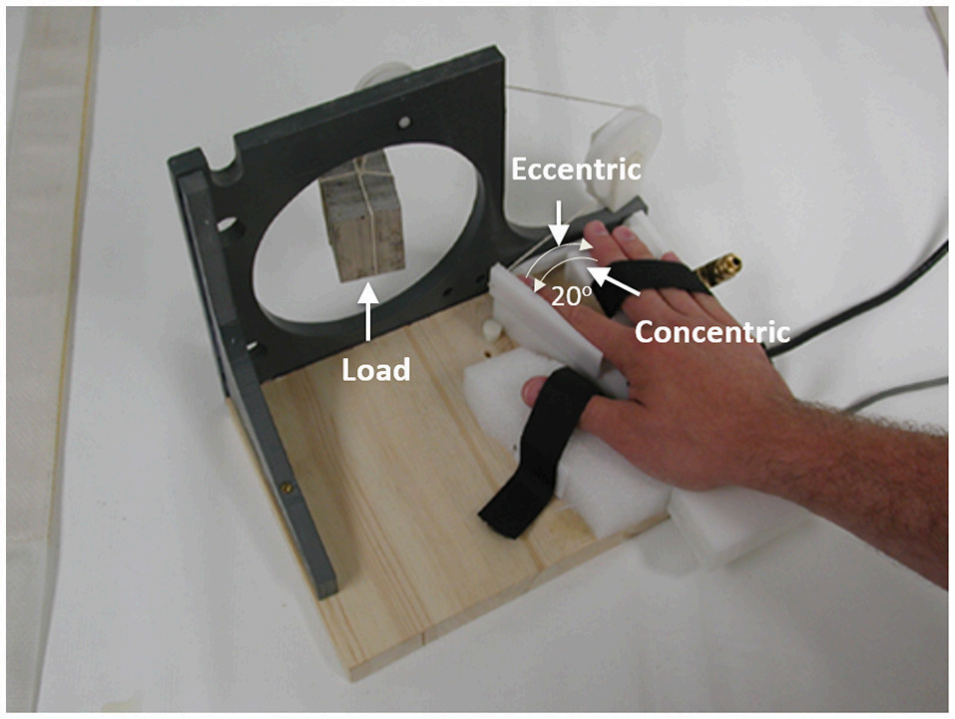
**An illustration of the experimental setup**. It shows a top view of the setup. As indicated by the two thin arrows in the left panel, the concentric contraction involved the index finger moved away from the middle finger (muscle shortening), in which the FDI muscle force was greater than the external resisting force, and the eccentric contraction involved the index finger moved toward the middle finger (muscle lengthening), which was a result of a greater external force than the force produced by the FDI muscle.

All subjects performed both concentric (muscle shortening, index finger moving toward the thumb) and eccentric (muscle lengthening, index finger moving away from the thumb) contractions of the first dorsal interosseous (FDI) muscle. Each type of contraction resulted in a 20° angular movement (a moveable lever on a self-built tool with a fixed 20° range of motion) from the initial position against a constant load with a constant speed (~10°/s or ~0.174 rad/s). A lead ball provided the load in 2.22 N increment and accounted for approximately 30% of maximal weight that the subject could lift with a concentric contraction of the FDI (The margin of error was less than 1.12 N). After a practicing block of 20 concentric contractions (CC) and 20 eccentric contractions (EC), a total of 20 CC and 20 EC testing trials were performed while functional brain images were taken. During the practice and testing, CC and EC were performed alternately (e.g., CC → EC → CC, etc.) while the subjects were lying supine in the MRI chamber with right hand resting prone on a wooden board. The right arm was abducted 10^0^ at shoulder joint with the elbow joint flexed to ~10^0^. The wrist, thumb, and other three (middle, ring, and little) fingers of the right hand were constrained. The right index finger was fastened to a movable lever attached to a load (30% maximal) through a non-elastic cable and a pulley fixed on the wooden board (Figure 1) which was put on the subject's abdominal area. The subjects performed CC of the right FDI muscle from the initial position (IP) by lifting the weight to the end position (EP) and EC from the EP by “lowering” the weight to the IP. A 10-s rest after both EC and CC movements was provided after each contraction during which the weight was supported by an external mechanism.

### Measurements and data processing

1. **Maximum Voluntary Contraction (MVC):** Isometric MVC force was measured by requesting the subjects maximally abduct their right index finger against an unmovable force transducer (Sensotec, Columbus, OH). Each subject performed two MVC trials and the trial with the higher peak force was chosen for further analysis. There was a 1-min rest between the two MVC trials.
2. **Movement Distance, Speed and Stability:** A custom-built, MRI-compatible goniometer for measuring the angular/movement distance and speed of the finger was attached to the movable lever. The movement data for both CC and EC were trigger-averaged across the 20 trials for each subject with a trigger signal, which was generated when the finger moved 2^0^ from the IP (10% of the movement distance) for CC and 2^0^ from the EP for EC (Fang et al., 2001, 2004). The movement speed was then derived from the trigger-averaged movement data. Standard deviation of the speed was taken to indicate stability of each movement.
3. **Image Acquisition:** The fMRI procedures (Gao et al., 1996; Yue et al., 2000; Yao et al., 2014) are designed to detect brain signal changes based on changes in brain blood oxygenation - the blood oxygenation level dependent (BOLD) signal changes. Functional brain images were acquired on a 3T Siemens Trio scanner (Siemens, Erlangen, Germany) using an 8-channel RF (Radio Frequency) head coil with a single shot gradient echo EPI (Echo Planar Imaging) pulse sequence (RT (Repetition Time) = 2 s, TE (Echo Time) = 30 ms, flip angle = 90°, slice thickness = 5 mm). Totally 21 slices were collected to cover the entire brain and cerebellum. The subjects were scanned in supine position and the head was padded to refrain from moving to minimize motion-related artifacts. Subjects were told to remain as still as possible during the entire scanning session. Structural images were collected using standard T1-weighted magnetization-prepared rapid acquisition gradient echo (MP-RAGE) sequence as follows: TR = 2200 ms; TE = 2.83 ms; flip angle = 13°; slice thickness = 1 mm. Totally 176 slices were acquired. All structural images were acquired after functional image acquisition. Both T1-weighted anatomical images and functional images were collected in the same transverse plane (aligned with a line connecting the anterior and posterior commissures). The image field of view and matrix for the anatomical images were 256 × 256 mm and 256 × 256, respectively; and those for the function images were 256 × 256 mm and 128 × 128, yielding an in-plane spatial resolution of 1 × 1 mm for anatomical images and 2 × 2 mm for functional images.

### Data analysis

The fMRI data processing was carried out using FEAT (FMRI Expert Analysis Tool) Version 5.98, part of FSL (FMRIB's Software Library, ww.fmrib.ox.ac.uk/fsl). The following pre-statistics processing was applied: motion correction using MCFLIRT (Jenkinson et al., 2002); non-brain removal using BET (Smith, 2002); spatial smoothing using a Gaussian kernel of FWHM (Full Width at Half Maximum) 5 mm; grand-mean intensity normalization of the entire 4D dataset by a single multiplicative factor; high-pass temporal filtering (Gaussian-weighted least-squares straight line fitting, with sigma = 50.0 s). Time-series data were fitted to a general linear model, in which BOLD signals from CC and EC contractions were modeled as boxcars convolved with hemodynamic function (HRF), using FILM (FMRIB's Improved Linear Model) with local autocorrelation correction. All fMRI data were first registered to high resolution structural images then to standard MNI space.

There are several ways to measure functional connectivity (FC), and a well-adopted approach is to calculate cross-correlation of an activation time course of a cortical area with the time course of a reference region or seed region. Our group has used this approach to investigate brain FC after muscle fatigue and demonstrated a significant increase in FC between the left and right primary motor cortices (Peltier et al., 2005) and between the primary motor cortex and multiple other cortical motor control regions during muscle fatigue (Peltier et al., 2005; Jiang et al., 2012). Thus, the seed-based FC approach was also adopted in this study. The seed region was defined by finding the 5 mm sphere centered at the maximally activated coordinate [−36, −24, 54] (in mm) in the left primary motor cortex (M1) as reported in the group activation map. Group level activation map was created using the same FEAT tool to localize the commonly activated brain regions during both CC and EC tasks. The seed time course was calculated by finding the mean of all time course data within the seed region. Subsequently, the FC maps were computed on voxel-based activation maps by finding the Pearson correlation coefficients between the data of the seed time course and those in the rest of the brain.

In order to evaluate the differences in FC maps between concentric and eccentric conditions, we constructed a paired *T*-test 2nd-level model in SPM Matlab toolbox (SPM8). Different contrasts were used to examine the mean FC map in both concentric and eccentric conditions as well as the difference between them in both directions (CC-EC and EC-CC).

## Results

All eleven subjects (23.25 ± 4.09 years old) successfully performed both concentric and eccentric contractions of the first dorsal interosseous (FDI) muscle as required. Both mechanical and fMRI data from the eleven subjects were further analyzed.

### MVC, movement speed and stability

The mean angular distance was 20^0^ for both CC and EC. The mean MVC forces were 48.9 ± 3.67 N. The mean speeds of EC and CC were 0.167 ± 0.015 rad/s and 0.174 ± 0.011 rad/s, respectively. The paired *t*-test on the movement speed was not significant between EC and CC, *t* (10) = −0.956, *P* = 0.362. The means of standard deviations of the speed, representing movement stability, for EC and CC were 0.038 and 0.035, respectively. The paired *t*-test on the movement stability was not significant between EC and CC, *t* (10) = 1.299, *P* = 0.22.

#### Functional connectivity map

**Figure 2:**
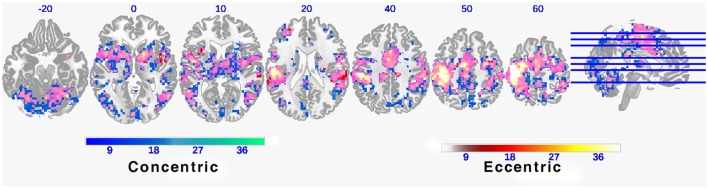
**Functional connectivity maps during concentric and eccentric contractions**. Axial slices of mean functional connectivity maps during concentric and eccentric muscle contractions (FEW corrected *p* < 0.01). The color bar indicates the raw *T*-value from the one sample *T*-test of the group mean of all 11 individual connectivity maps. Large overlapping areas of both connectivity maps can be observed (indicated by pink clusters).

As expected, both CC and EC tasks have elicited brain activation in a wide range of motor control-related regions including bilateral M1, primary sensory cortex (S1), supplementary motor area (SMA), and cerebellum. A majority of these activated brain regions are shared by the two tasks (clusters colored in pink in Figure 2). However, it is worth noting that there are more and greater clusters in terms of size in mean FC map for CC than that for EC despite that the magnitude of brain activation was greater in EC than CC.

**Figure 3:**
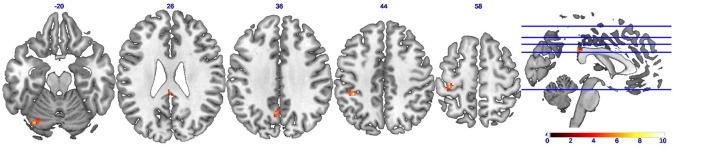
**Brain regions showing strengthened functional connectivity during concentric contractions compared to eccentric contractions (CC-EC)**. Axial slices of brain clusters showing higher functional connectivity (uncorrected *p* < 0.01) during concentric than eccentric muscle contractions. The color bar indicates the raw *T*-value from the paired sample *T*-test. A wide spread regions including the left supplementary motor cortex (SMA) and cerebellum were found to have strengthened connectivity with the seed region (left M1).

A statistical comparison of all FC maps during the CC task with maps during the EC task with contrast set as CC-EC revealed detailed spatial information on the brain regions with higher FC in CC than in EC. As shown in Figure 3 and Table 1, a number of regions including left SMA and cerebellum were found to have significantly (uncorrected *p* < 0.01) strengthened connectivity with the seed region (left M1) in CC compared to EC.

**Table 1:** **Brain regions with strengthened FC during CC compared to EC**.

**Region**	***X* (mm)**	***Y* (mm)**	***Z* (mm)**	**Volume (mm^3^)**
Left inferior parietal	−38	−36	44	144
Left cerebelum	−28	−72	−22	408
Left post central	−36	−26	56	144
Posterior cingulate gyrus	0	−34	28	128
Left precuneus	−2	−60	36	88
Right parietal lobe	16	−56	64	88

**Figure 4:**
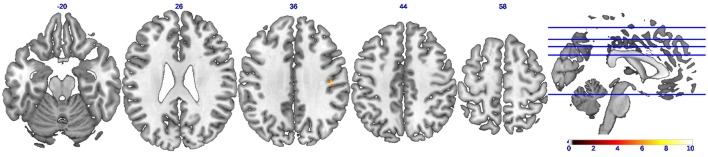
**Brain regions showing strengthened functional connectivity during eccentric contractions compared to concentric contractions (EC-CC)**. Axial slices showing only ONE brain cluster with higher functional connectivity (uncorrected *p* < 0.01) during eccentric muscle contractions than concentric muscle contractions. The color bar indicates the raw *T*-value from the paired sample *T*-test.

A statistical comparison of all FC maps during CC task with maps during EC tasks with contrast set as EC-CC revealed detailed spatial information on the brain regions with higher FC in EC than in CC. As shown in Figure 4 and Table 2, only a cluster in the right postcentral gyrus was found to have significantly (uncorrected *p* < 0.01) strengthened connectivity with the seed region (left M1) during EC compared to CC.

**Table 2:** **Brain regions with strengthened FC during EC compared to CC**.

**Region**	***X* (mm)**	***Y* (mm)**	***Z* (mm)**	**Volume (mm^3^)**
Right postcentral	44	−16	38	80

## Discussion

The purpose of this study was to determine if brain functional connectivity (FC) would be different for controlling concentric (CC) than eccentric (EC) right hand FDI contractions. Gaining this knowledge is important for several reasons. For example, EC movements are more complex and difficult to control than CC, which poses increased chances of injuries such as falls during walking downstairs (EC of hip and knee extensors, and ankle plantar flexors) especially in vulnerable populations such as frail patients and elderly individuals. Previous studies have shown differences in brain activation between CC and EC such as greater fMRI (Yao et al., 2014) and EEG-derived MRCP (Fang et al., 2001, 2004) signals. However, the current study is the first to show FC differences between CC and EC of the FDI. A better understanding of neural mechanisms underlying different types of human movements would potentially help gain insights into the mechanisms of neuromuscular injuries and design more targeted therapeutic programs to prevent or reduce chances of such injuries.

Based on our best knowledge, this is the first study to examine FC based on fMRI data collected during CC and EC. The novel finding of this study is that CC is associated with significantly stronger FC than EC although the patterns of FC map for CC and EC are similar (See Figure 1). Fang and his colleagues conducted two studies to monitor magnitude of cortical potential derived from electroencephalography (EEG) signals at submaxial (Fang et al., 2001) and maximal (Fang et al., 2004) intensity levels of CC and EC. They found that although the elbow flexor muscle activities (EMG) were lower during EC than CC, the magnitude of movement-related cortical potential (MRCP) derived from the EEG recordings was significantly greater for EC than CC at both intensity levels. Yao et al. (2014) and Kwon and Park (2011) also found significantly greater activities in motor cortical areas such as M1, SMA, and premotor cortex during EC than CC. At the first glance, it seems that the current finding is contradictory to previous observations (Fang et al., 2001, 2004; Kwon and Park, 2011; Yao et al., 2014) that have consistently shown significantly greater activities in motor cortical areas during EC than CC. However, it should be noted that FC and magnitude of cortical activation are two types of measurements and not directly comparable. The aforementioned studies (Fang et al., 2001, 2004; Kwon and Park, 2011; Yao et al., 2014) were targeted on determining the number and/or intensity of activated cortical areas, or amplitude of cortical signal associated either with CC and/or with EC. In contrast, the current study was aimed at examining the strength of the relationship (i.e., FC) between the activated cortical areas with a seed region (left M1 in the current study). Thus, there are no conflicts between the findings of the aforementioned investigations and the current study.

Rather than contradictory to each other, the findings from the current study (stronger FC for CC than EC) and from the previous studies (higher cortical activation volume and amplitude for EC than CC) may be explained by the reasoning that the stronger brain activation (larger volume and higher amplitude) for EC is necessary to “compensate” for weaker functional connectivity among areas in the motor control network. On the other hand, because of stronger FC, controlling a CC does not need to recruit as many neural populations in the brain and raise their activation level as high as for an EC. Consequently the brain control of a CC is more efficient than an EC. Such efficiency may be the result of learning and/or adaptation since people use more CC than EC and they pay more attention when performing CC during their daily life and, thus, have better control of CC than EC. Alternatively, since most of CC movements are anti-gravity but EC activities are with gravity, these differences may also contribute to the notion of “control efficiency.” Regarding the relationship between FC and motor skill learning and between FC and efficiency of motor movements, literature has shown a positive relationship between the two. That is, advancement in motor skill learning is associated with dynamical neural changes including the increase in FC in related cortical areas (Ungerleider et al., 2002). For example, in a study aimed at obtaining efficiency of a novel complex movement, McNamara et al. (2007) had their subjects to learn fast synchronous co-contractions of abductor pollicis brevis and deltoid muscles. They found that the learned synchrony of muscle contractions was highly associated with the significantly increased FC. This result indicates that establishing FC between and among motor cortical areas is a crucial process underlying learning new motor skills. Most recently, Zhang et al. (2014) also demonstrated a positive relationship between FC and motor skill learning by using mental imagery practice.

Putting together, the findings from the current study along with previous observations regarding the relationship of FC and motor skill learning and movement efficiency have significant meanings for therapeutic and sports-training settings in terms of providing practitioners such as physical therapists and coaches with neural base for assessing learning process and/or for planning exercise routines. Previous studies have consistently shown poorer performance during EC movements than CC movements, which is more true for aging population than young adults (Laidlaw et al., 1999; Fang et al., 2004; Yao et al., 2014). And such lack of movement accuracy in EC could be caused by the lack of FC in the motor control network. To overcome this limitation, the nervous system may need to use more resources such as the number and intensity of activated cortical areas to accomplish an EC. Practice/learning should be able to improve movement accuracy of EC and FC could serve as a measurement to judge the effectiveness of a training strategy designed for improving EC movements or other complex movements. The challenge for future work is to discover if same or similar FC patterns will be found when the maximum loads for both EC and CC are used, and/or the non-dominant hand is tested.

## Summary

The mechanical data analyses indicate no significant differences in mean MVC forces, speeds, and movement stability between CC and EC. The current study also applied functional connectivity (FC) analysis to examine fMRI brain signal time course data during concentric muscle contractions (CC) and eccentric muscle contractions (EC). The major finding in this analysis is that the CC is associated with significantly stronger FC than EC in a number of cortical fields with the M1. This finding may indicate that CC is a better learned/adapted movement than EC, or the antigravity nature of CC may require stronger FC to accomplish the movement. The finding (i.e., greater FC for CC than EC) advances our understanding of motor control mechanisms and provides useful information for guiding movement rehabilitation and sports training.

## Author contributions

WY: Contributed to experimental design, data collection and analysis, and manuscript writing; ZJ: Contributed to data analysis and manuscript writing; JL: Contributed to data collection/analysis and manuscript writing; CJ: Contributed to data analysis and manuscript writing; CF: Contributed to data collection/analysis. JLL: Contributed to experimental design and data analysis; YH: Contributed to experimental design and data analysis; GY: Contributed to experimental design and manuscript writing.

## Funding

This study was supported in part by San Antonio Life Science Institutes.

### Conflict of interest statement

The authors declare that the research was conducted in the absence of any commercial or financial relationships that could be construed as a potential conflict of interest.
